# Long noncoding RNA *NR2F1-AS1* enhances the malignant properties of osteosarcoma by increasing forkhead box A1 expression via sponging of microRNA-483-3p

**DOI:** 10.18632/aging.102563

**Published:** 2019-12-04

**Authors:** Shenglong Li, Ke Zheng, Yi Pei, Wei Wang, Xiaojing Zhang

**Affiliations:** 1 Department of Bone and Soft Tissue Tumor Surgery, Cancer Hospital of China Medical University, Liaoning Cancer Hospital and Institute, Shenyang, Liaoning 110042, P.R. China

**Keywords:** NR2F1-AS1, microRNA-483-3p, forkhead box A1, osteosarcoma therapy

## Abstract

The long noncoding RNA *NR2F1-AS1* has been found to promote the development of hepatocellular carcinoma and endometrial cancer. In this study, we measured *NR2F1-AS1* expression in osteosarcoma (OS), determined the involvement of *NR2F1-AS1* in the malignant properties of OS, and investigated the underlying mechanisms. *NR2F1-AS1* was found to be upregulated in OS tumors and cell lines. The increased *NR2F1-AS1* level was closely associated with advanced clinical stage and distant metastasis in patients with OS. Patients with OS in an *NR2F1-AS1* high-expression group demonstrated significantly shorter overall survival than did patients in an *NR2F1-AS1* low-expression group. *NR2F1-AS1* knockdown inhibited OS cell proliferation, migration, and invasion and promoted cell cycle arrest and apoptosis *in vitro* and slowed tumor growth *in vivo*. *NR2F1-AS1* was found to function as a competing endogenous RNA by directly sponging microRNA-483-3p (miR-483-3p) and upregulating its target oncogene forkhead box A1 (*FOXA1*). Finally, rescue experiments revealed that knockdown of miR-483-3p and recovery of FOXA1 expression both attenuated the influence of the *NR2F1-AS1* knockdown on OS cells. Thus, *NR2F1-AS1* plays an oncogenic role in OS through sponging miR-483-3p and thereby upregulating FOXA1, suggesting an additional target for osteosarcoma therapeutics.

## INTRODUCTION

Osteosarcoma (OS) is a highly malignant bone cancer that ranks as the most common primary human malignant tumor among children and adolescents [1]. OS generally occurs in the metaphysis of a long bone [2]. Globally, the prevalence of OS is ~4.4 individuals per million [3]. Current standard treatment is surgical resection followed by adjuvant and neoadjuvant chemotherapy [4]. Considerable progress in diagnosis and therapy has notably improved the clinical outcomes of patients with OS [5]; unfortunately, their long-term prognosis is still unfavorable, with a 5-year survival rate of less than 30% [6]. Therefore, elucidation of the molecular events behind the formation and progression of OS is urgently needed for identifying novel therapeutic techniques and improving the prognosis of the patients with this disease.

Long noncoding RNAs (lncRNAs) are a group of RNAs over 200 nucleotides in length [7]. Although lncRNAs cannot be translated into proteins, they can modulate a variety of pathological and physiological processes by transcriptional and/or post-transcriptional regulation [8, 9]. LncRNAs play tumor-suppressive or oncogenic roles in tumorigenesis and tumor progression through multiple molecular mechanisms, including epigenetic silencing, mRNA splicing, and interactions with microRNAs (miRNAs), proteins, or messenger RNAs (mRNAs) [10, 11]. In the field of OS research, many lncRNAs have been reported to be upregulated or downregulated in OS and to exert crucial effects on oncogenicity [12–14]. Accordingly, further research into the expression and specific functions of lncRNAs in OS will improve the diagnosis, prognosis, treatment, and prevention of OS.

MicroRNAs (miRNAs, miRs) are short single-stranded noncoding RNA molecules [15]. They participate in gene silencing and repress gene expression by directly interacting with the 3′ untranslated region (3′-UTRs) of target mRNAs, resulting in mRNA degradation and/or repression of translation [16]. MiRNAs are implicated in diverse biological and pathological processes, including embryonic development, metabolism, and carcinogenesis [17]. Several crucial activities of miRNAs in OS have been reported [18–20]. For instance, miR-217 [21] and miR-337 [22] are weakly expressed in OS and function as tumor suppressors. By contrast, the expression of miR-522 [23], miR-765 [24], and miR-889 [25] is high in OS, and these miRNAs act as oncogenic molecules. Therefore, miRNAs may serve as attractive therapeutic targets in OS.

A novel oncogenic lncRNA, *NR2F1-AS1*, has been reported to promote the growth of hepatocellular carcinoma [26] and endometrial cancer [27]. By contrast, to the best of our knowledge, the expression and detailed functions of *NR2F1-AS1* in OS remain poorly studied. Therefore, we attempted to quantify *NR2F1-AS1* levels in OS tumors and cell lines, determine its function in OS progression, and investigate its mechanism of action. These data may help to develop methods for the early diagnosis of OS and to identify effective therapeutic targets.

## RESULTS

### The expression of *NR2F1-AS1* is high in OS tissue samples and cell lines and correlates with poor clinical outcomes

**Figure 1:**
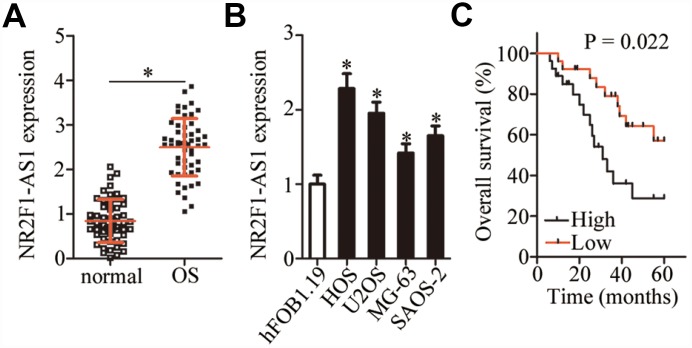
***NR2F1-AS1* expression in OS tissue samples and cell lines.** (**A**) *NR2F1-AS1* expression in 53 pairs of OS tissue samples and adjacent normal tissues was analyzed by RT-qPCR. *P < 0.05 vs. adjacent normal tissues. (**B**) The expression of *NR2F1-AS1* in four human OS cell lines (HOS, U2OS, MG-63, and SAOS-2) and normal osteoblasts (hFOB1.19) was tested by RT-qPCR. *P < 0.05 vs. hFOB1.19 cells. (**C**) Correlation between *NR2F1-AS1* expression and overall survival of patients with OS was determined by Kaplan–Meier analysis; n = 53, P = 0.022.

*NR2F1-AS1* expression in 53 pairs of OS tissue samples and adjacent normal tissues was measured by RT-qPCR. The data showed markedly higher *NR2F1-AS1* expression in OS tissue samples relative to the adjacent normal tissue samples (Figure 1A, P < 0.05). The expression of *NR2F1-AS1* in four human OS cell lines (HOS, U2OS, MG-63, and SAOS-2) and normal osteoblasts (hFOB1.19) was also examined by RT-qPCR. *NR2F1-AS1* was upregulated in all four OS cell lines compared with hFOB1.19 cells (Figure 1B, P < 0.05).

The 53 patients with OS were classified into either an *NR2F1-AS1* high-expression group or *NR2F1-AS1* low-expression group based on the median value (2.55) of *NR2F1-AS1* expression among the OS tissue samples as determined by RT-qPCR. Higher *NR2F1-AS1* expression significantly correlated with more advanced clinical stage (P = 0.024) and distant metastasis (P = 0.042) among the 53 patients with OS (Table 1). In addition, patients with OS in the *NR2F1-AS1* high-expression group demonstrated shorter overall survival than did the patients in the *NR2F1-AS1* low-expression group (Figure 1C, P = 0.022). These results indicated that *NR2F1-AS1* might be closely associated with the malignancy of OS.

**Table 1:** Association between NR2F1-AS1 expression and clinical parameters of patients with OS.

**Clinical parameters**	**NR2F1-AS1 expression**		**P**
	**High (n=27)**	**Low (n=26)**	
Age (years)			0.293
<18	20 (74.1%)	23 (88.5%)	
≥18	7 (25.9%)	3 (11.5%)	
Gender			0.782
Male	17 (63.0%)	15 (57.7%)	
Female	10 (37.0%)	11 (42.3%)	
Tumor size (cm)			0.569
< 5	16 (59.3%)	18 69.2%)	
≥ 5	11 (40.7%)	8 (30.8%)	
Clinical staging			0.024^*^
I-II	12 (44.4%)	20 (76.9%)	
III	15 (55.6%)	6 (23.1%)	
Distant metastasis			0.042^*^
Present	14 (51.9%)	21 (80.8%)	
Absent	13 (48.1%)	5 (19.2%)	

### Silencing of *NR2F1-AS1* suppresses the malignant properties of OS cells

**Figure 2:**
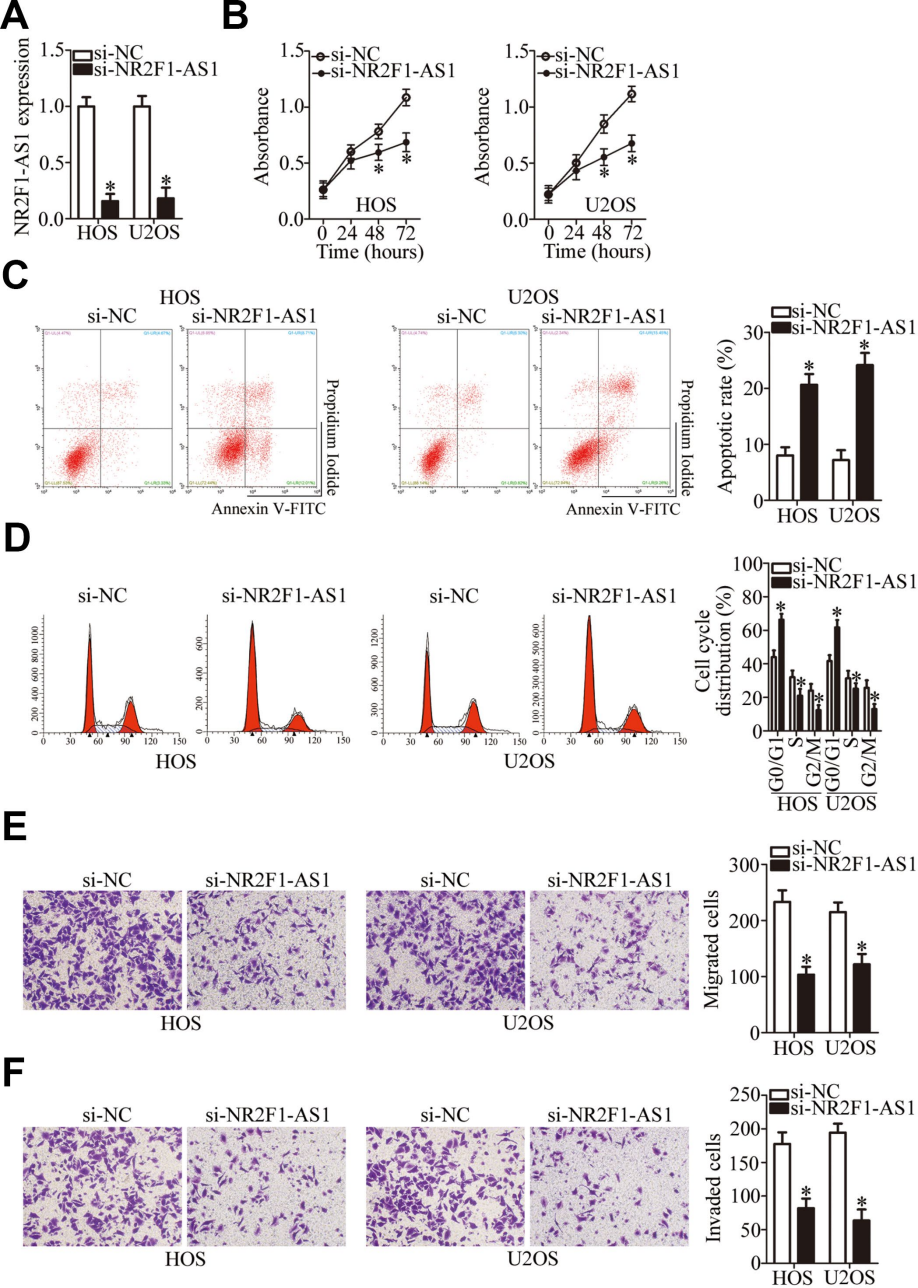
***NR2F1-AS1* silencing inhibits the proliferation, migration, and invasiveness and promotes the apoptosis of HOS and U2OS cells.** (**A**) Either si-NR2F1-AS1 or si-NC was transfected into HOS and U2OS cells. At 48 h after transfection, RT-qPCR analysis was performed to assess the transfection efficiency. *P < 0.05 vs. group “si-NC.” (**B**) The CCK-8 assay result showing cell proliferation status under the influence of the *NR2F1-AS1* knockdown in HOS and U2OS cells. *P < 0.05 vs. the si-NC group. (**C**) The apoptotic rate of HOS and U2OS cells after transfection with either si-NR2F1-AS1 or si-NC was detected by means of an Annexin V–FITC Apoptosis Detection Kit. *P < 0.05 vs. group si-NC. (**D**) Flow cytometry was carried out to examine the cell cycle status of HOS and U2OS cells after transfection with either si-NR2F1-AS1 or si-NC. *P < 0.05 vs. group si-NC. (**E**, **F**) Transwell migration and invasion assays quantified the migratory and invasive abilities of HOS and U2OS cells after the transfection of either si-NR2F1-AS1 or si-NC. *P < 0.05 vs. group si-NC.

The HOS and U2OS cell lines manifested higher *NR2F1-AS1* expression compared with the other two OS cell lines (MG-63 and SAOS-2); therefore, these two cell lines were selected for further study. To determine the participation of *NR2F1-AS1* in OS progression, an siRNA targeting *NR2F1-AS1* was utilized for silencing endogenous *NR2F1-AS1* expression in HOS and U2OS cells. RT-qPCR confirmed the efficient knockdown of *NR2F1-AS1* in these cells after transfection with si-NR2F1-AS1 (Figure 2A, P < 0.05).

A CCK-8 assay was then performed to determine the effect of *NR2F1-AS1* knockdown on OS cell proliferation, which showed that this knockdown attenuated the proliferative ability of HOS and U2OS cells (Figure 2B, P < 0.05). Apoptosis of the si-NR2F1-AS1–transfected or si-NC–transfected HOS and U2OS cells was analyzed by flow cytometry. The results indicated that the knockdown of *NR2F1-AS1* promoted the apoptosis of HOS and U2OS cells (Figure 2C, P < 0.05). Analysis of the cell cycle distribution showed that after si-NR2F1-AS1 transfection, the proportion (%) of HOS and U2OS cells at the G0–G1 transition greatly increased, whereas the proportion of cells in the S phase decreased (Figure 2D, P < 0.05). Consequently, the inhibition of OS cell proliferation by *NR2F1-AS1* knockdown could be attributed to the induction of apoptosis and promotion of cell cycle arrest.

To test whether *NR2F1-AS1* knockdown affects OS cell migration and invasion, transwell migration and invasion assays were performed on *NR2F1-AS1*–deficient HOS and U2OS cells. Knockdown of *NR2F1-AS1* yielded an obvious decrease in HOS and U2OS cell migration (Figure 2E, P < 0.05) and invasion (Figure 2F, P < 0.05). Altogether, these results suggested that *NR2F1-AS1* knockdown repressed the growth and metastasis of OS cells *in vitro*.

### *NR2F1-AS1* acts as a competing endogenous RNA (ceRNA) on miR-483-3p in OS cells

**Figure 3:**
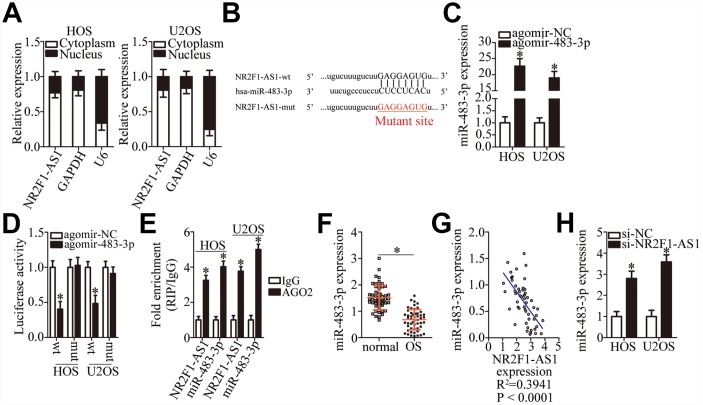
**MiR-483-3p is a target of *NR2F1-AS1* in OS cells.** (**A**) Nuclear/cytoplasmic fractionation analysis of *NR2F1-AS1* expression in HOS and U2OS cells. (**B**) A diagram of the wild-type and mutant binding sites for miR-483-3p in *NR2F1-AS1*. (**C**) RT-qPCR analysis was performed to determine miR-483-3p expression in HOS and U2OS cells that were transfected with either agomir-483-3p or agomir-NC. *P < 0.05 vs. group agomir-NC. (**D**) Either NR2F1-AS1-wt or NR2F1-AS1-mut was cotransfected with either agomir-483-3p or agomir-NC into HOS and U2OS cells. The luciferase reporter assay was performed at 48 h post-transfection to measure luciferase activity. *P < 0.05 vs. the agomir-NC group. (**E**) The RIP assay was conducted to evaluate the direct interaction between agomir-483-3p and *NR2F1-AS1*. Agomir-483-3p and *NR2F1-AS1* were both immunoprecipitated by the anti-AGO2 antibody from the lysates of HOS and U2OS cells. *P < 0.05 vs. the IgG group. (**F**) RT-qPCR was carried out to determine miR-483-3p expression in the 53 pairs of OS tissue samples and the adjacent normal tissues. *P < 0.05 vs. adjacent normal tissues. (**G**) The correlation between miR-483-3p and *NR2F1-AS1* expression levels among the 53 OS tissue samples was determined via Spearman’s correlation analysis. R^2^ = 0.3941, P < 0.0001. (**H**) The expression of miR-483-3p in HOS and U2OS cells transfected with either si-NR2F1-AS1 or si-NC was measured by RT-qPCR. *P < 0.05 vs. group si-NC.

To investigate the molecular mechanisms underlying the *NR2F1-AS1* functions in OS cells, a nuclear/cytoplasmic fractionation experiment was performed, which revealed that *NR2F1-AS1* was mainly located in the cytoplasm of OS cells (Figure 3A), suggesting that this lncRNA may act as an miRNA sponge in OS cells. After screening for miRNAs using the online software starBase 3.0, miR-483-3p was predicted to be a potential target of *NR2F1-AS1* (Figure 3B). MiR-483-3p was chosen for further experimental validation because of its known tumor-suppressive actions in multiple types of human cancers [28–30]. To verify this, a luciferase reporter assay was carried out in HOS and U2OS cells after cotransfection with either NR2F1-AS1-wt or NR2F1-AS1-mut and either agomir-483-3p or agomir-NC. The results showed that agomir-483-3p transection, which significantly increased miR-483-3p expression (Figure 3C, P < 0.05), reduced the luciferase activity of NR2F1-AS1-wt (P < 0.05). In contrast, the luciferase activity of NR2F1-AS1-mut was not affected by miR-483-3p overexpression in HOS and U2OS cells (Figure 3D). Additionally, results from a RIP assay of HOS and U2OS cells indicated that *NR2F1-AS1* was preferentially enriched on AGO2-containing beads (Figure 3E, P < 0.05), suggesting that miR-483-3p is a target of *NR2F1-AS1*.

RT-qPCR was carried out to determine the expression of miR-483-3p in OS tissue samples and assess the correlation between miR-483-3p and *NR2F1-AS1* expression levels in OS tumors. RT-qPCR indicated that the expression of miR-483-3p was low in OS tissue samples compared with the adjacent normal tissues (Figure 3F, P < 0.05). Next, an inverse correlation between miR-483-3p and *NR2F1-AS1* expression was demonstrated in these OS tissue samples (Figure 3G; R^2^ = 0.3941, P < 0.0001). The effects of *NR2F1-AS1* knockdown on miR-483-3p expression in HOS and U2OS cells were then determined using RT-qPCR. As shown in Figure 3H, the knockdown of *NR2F1-AS1* increased the amount of miR-483-3p in HOS and U2OS cells (P < 0.05), suggesting that *NR2F1-AS1* inhibits miR-483-3p expression in OS cells. Collectively, these results suggest that *NR2F1-AS1* functions as a ceRNA of miR-483-3p in OS cells.

### *FOXA1* mRNA is a direct target of miR-483-3p in OS cells

**Figure 4:**
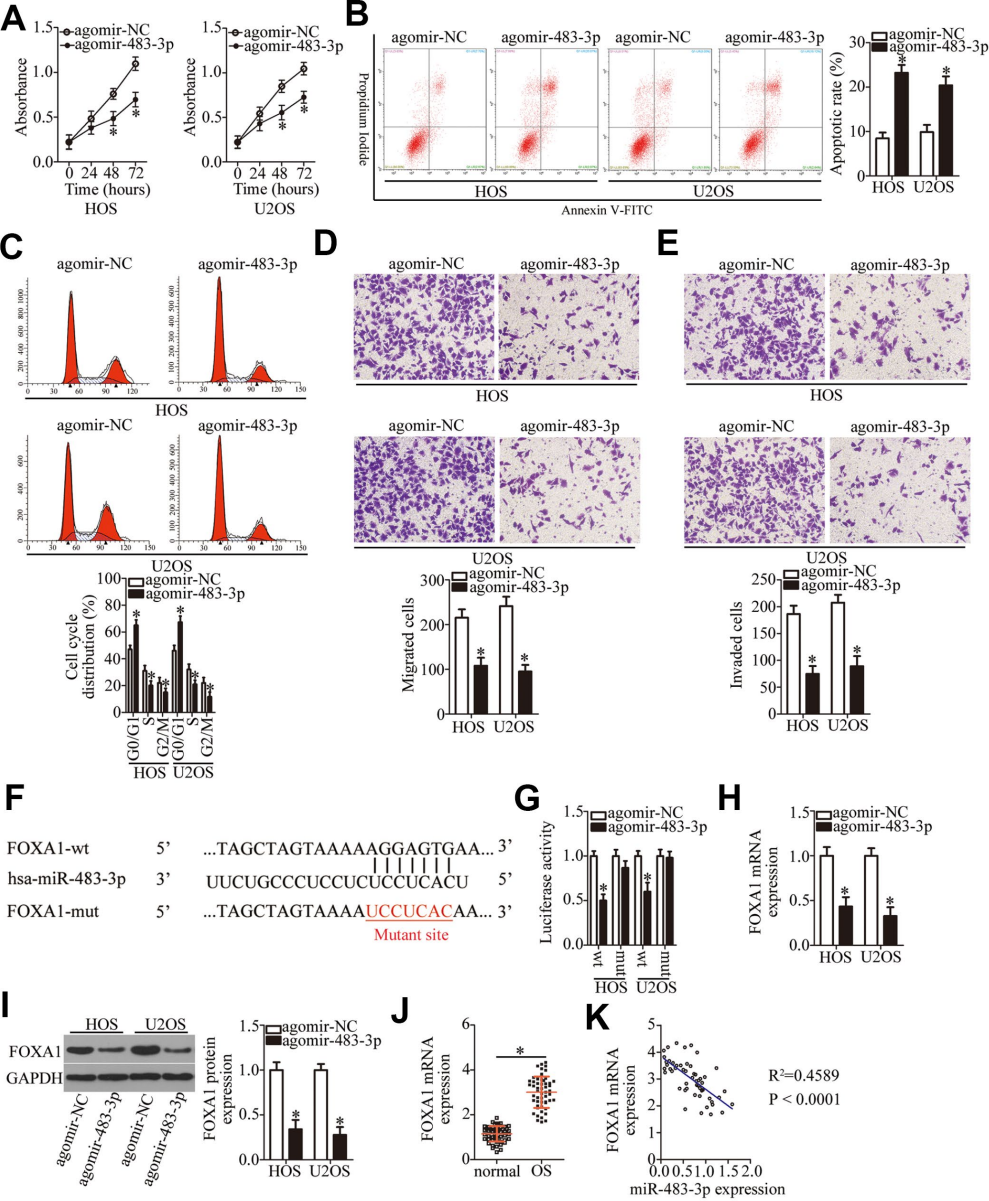
**MiR-483-3p directly targets *FOXA1* mRNA and plays tumor-suppressive roles in OS cells**. HOS and U2OS cells were transfected with either agomir-483-3p or agomir-NC. The transfected cells were studied in functional experiments. (**A**) The proliferative ability of miR-483-3p–overexpressing HOS and U2OS cells was tested by the CCK-8 assay. *P < 0.05 vs. the agomir-NC group. (**B**, **C**) The apoptosis rate and cell cycle status of HOS and U2OS cells were determined via flow-cytometric analysis after either agomir-483-3p or agomir-NC transfection. *P < 0.05 vs. the agomir-NC group. (**D**, **E**) Transwell migration and invasion assays were used to assess the impact of miR-483-3p overexpression on the migration and invasiveness of HOS and U2OS cells. *P < 0.05 vs. the agomir-NC group. (**F**) The predicted miR-483-3p–binding site in the 3′-UTR of the *FOXA1* mRNA. The mutated binding sequence is also shown. (**G**) Either FOXA1-wt or FOXA1-mut along with either agomir-483-3p or agomir-NC was introduced into HOS and U2OS cells. After 48 h of transfection, firefly luciferase activity was measured and normalized to that of Renilla luciferase. *P < 0.05 vs. the agomir-NC group. (**H**, **I**) The mRNA and protein levels of FOXA1 in HOS and U2OS cells that were transfected with either agomir-483-3p or agomir-NC were respectively examined by RT-qPCR and western blotting. *P < 0.05 vs. the agomir-NC group. (**J**) The expression of FOXA1 mRNA in the 53 pairs of OS tissue samples and the adjacent normal tissues was tested via RT-qPCR analysis. *P < 0.05 vs. adjacent normal tissues. (**K**) The expression correlation between miR-483-3p and *FOXA1 mRNA* in the 53 OS tissues was analyzed through Spearman’s correlation analysis. R^2^ = 0.4589, P < 0.0001.

We next examined the roles of miR-483-3p in OS and elucidated the molecular mechanisms underlying the activities of miR-483-3p in OS cells. HOS and U2OS cells were transfected with either agomir-483-3p or agomir-NC, and the influence of miR-483-3p overexpression on OS cells was evaluated. The ectopic miR-483-3p expression decreased HOS and U2OS cell proliferation (Figure 4A, P < 0.05), induced cell apoptosis (Figure 4B, P < 0.05), and promoted cell cycle arrest (Figure 4C, P < 0.05). In addition, ectopic miR-483-3p expression reduced the migratory (Figure 4D, P < 0.05) and invasive (Figure 4E, P < 0.05) abilities of HOS and U2OS cells, as evidenced by transwell migration and invasion assays.

Two bioinformatics tools, miRDB and TargetScan 7.1, were used to predict the potential target of miR-483-3p. As illustrated in Figure 4F, the 3′-UTR of *FOXA1* mRNA contains a 7-bp complementary sequence that may directly interact with miR-483-3p. A luciferase reporter assay was performed to confirm that miR-483-3p can directly bind to the 3′-UTR of *FOXA1* mRNA. The results suggested that the luciferase activity of HOS and U2OS cells transfected with FOXA1-wt was restrained by miR-483-3p overexpression (P < 0.05). In contrast, the luciferase activity of FOXA1-mut was unaffected by agomir-483-3p cotransfection (Figure 4G). RT-qPCR and western blotting were then performed to measure the levels of FOXA1 mRNA and protein in the HOS and U2OS cells that were transfected with either agomir-483-3p or agomir-NC. The data showed that ectopic miR-483-3p expression significantly decreased the expression of FOXA1 in HOS and U2OS cells at both the mRNA (Figure 4H, P < 0.05) and protein levels (Figure 4I, P < 0.05). Furthermore, RT-qPCR analysis indicated that *FOXA1* mRNA expression was higher in OS tissue samples than in the adjacent normal tissues (Figure 4J, P < 0.05). Furthermore, *FOXA1* expression was negatively correlated with the miR-483-3p level (Figure 4K; R^2^ = 0.4589, P < 0.0001). As a whole, these results confirmed the role of miR-483-3p as a tumor-suppressive miRNA in OS and identified *FOXA1* as a direct target gene of miR-483-3p in OS.

### *NR2F1-AS1* exerts oncogenic action on OS progression via the miR-483-3p–FOXA1 axis

**Figure 5:**
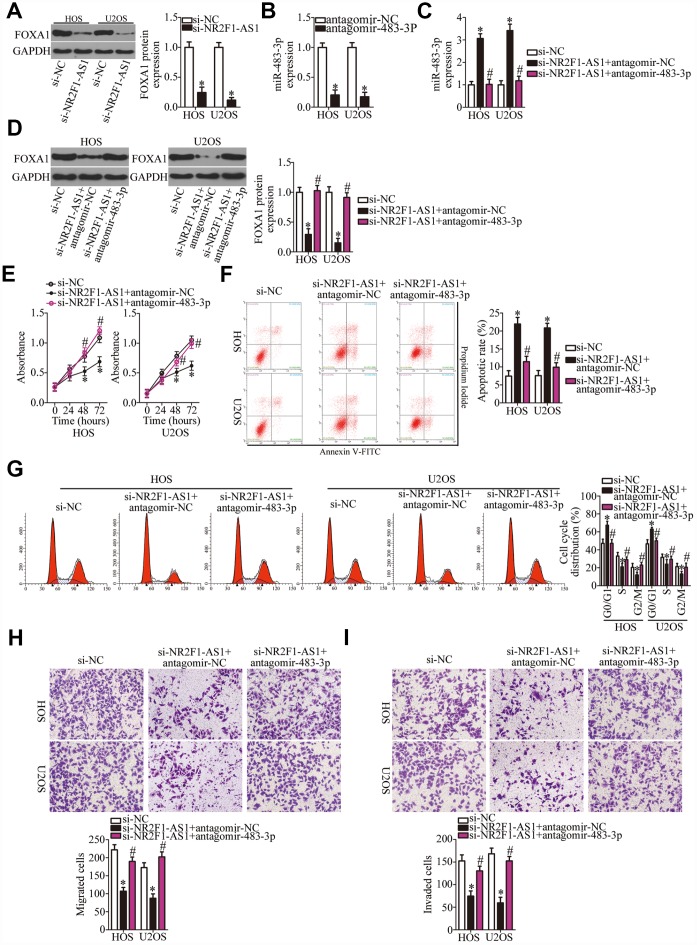
**Inhibition of miR-483-3p weakens the effects of *NR2F1-AS1* knockdown in OS cells.** (**A**) Western blotting analysis of FOXA1 expression in *NR2F1-AS1*–deficient HOS and U2OS cells. *P < 0.05 vs. the si-NC group. (**B**) HOS and U2OS cells were transfected with either antagomir-483-3p or antagomir-NC. After 48 h of transfection, total RNA was isolated from the cells and used for quantitation of miR-483-3p. *P < 0.05 vs. group antagomir-NC. (**C**, **D**) Si-NR2F1-AS1 along with either antagomir-483-3p or antagomir-NC was introduced into HOS and U2OS cells. The expression levels of miR-483-3p and of the FOXA1 protein in the transfected cells were respectively measured by RT-qPCR and western blotting. *P < 0.05 vs. the si-NC group. ^#^P < 0.05 vs. group si-NR2F1-AS1+antagomir-NC. (**E**–**I**) The proliferation, apoptotic rate, cell cycle distribution, migration, and invasiveness of the aforementioned cells were determined by the CCK-8 assay, flow cytometry, and transwell migration and invasion assays, respectively. The decrease in cell proliferation, increase in apoptosis, cell cycle arrest, and inhibition of cell migration and invasion were partially reversed by miR-483-3p knockdown. *P < 0.05 vs. group si-NC. ^#^P < 0.05 vs. group si-NR2F1-AS1+antagomir-NC.

As mentioned above, *FOXA1* was validated as a direct target gene of miR-483-3p in OS cells; accordingly, we next tested whether *NR2F1-AS1* is implicated in the regulation of *FOXA1* expression in OS cells. The protein level of FOXA1 was significantly lower in the si-NR2F1-AS1–transfected HOS and U2OS cells, as revealed by western blotting (Figure 5A, P < 0.05). Therefore, we speculated that the miR-483-3p–FOXA1 axis may play a role in the effects of *NR2F1-AS1* on the malignant characteristics of OS cells. To verify this prediction, HOS and U2OS cells were transfected with si-NR2F1-AS1 along with either antagomir-483-3p or antagomir-NC. First, the efficiency of antagomir-483-3p transfection was confirmed via RT-qPCR (Figure 5B, P < 0.05). The increase in miR-483-3p levels (Figure 5C, P < 0.05) and the decrease in FOXA1 protein amounts (Figure 5D, P < 0.05) resulting from *NR2F1-AS1* knockdown were reversed by antagomir-483-3p cotransfection of HOS and U2OS cells. Then, a series of functional experiments was performed on the cotransfected cells. Inhibition of miR-483-3p expression attenuated the influence of *NR2F1-AS1* knockdown on the proliferation (Figure 5E, P < 0.05), apoptosis (Figure 5F, P < 0.05), cell cycle distribution (Figure 5G, P < 0.05), migration (Figure 5H, P < 0.05), and invasiveness (Figure 5I, P < 0.05) of HOS and U2OS cells.

**Figure 6:**
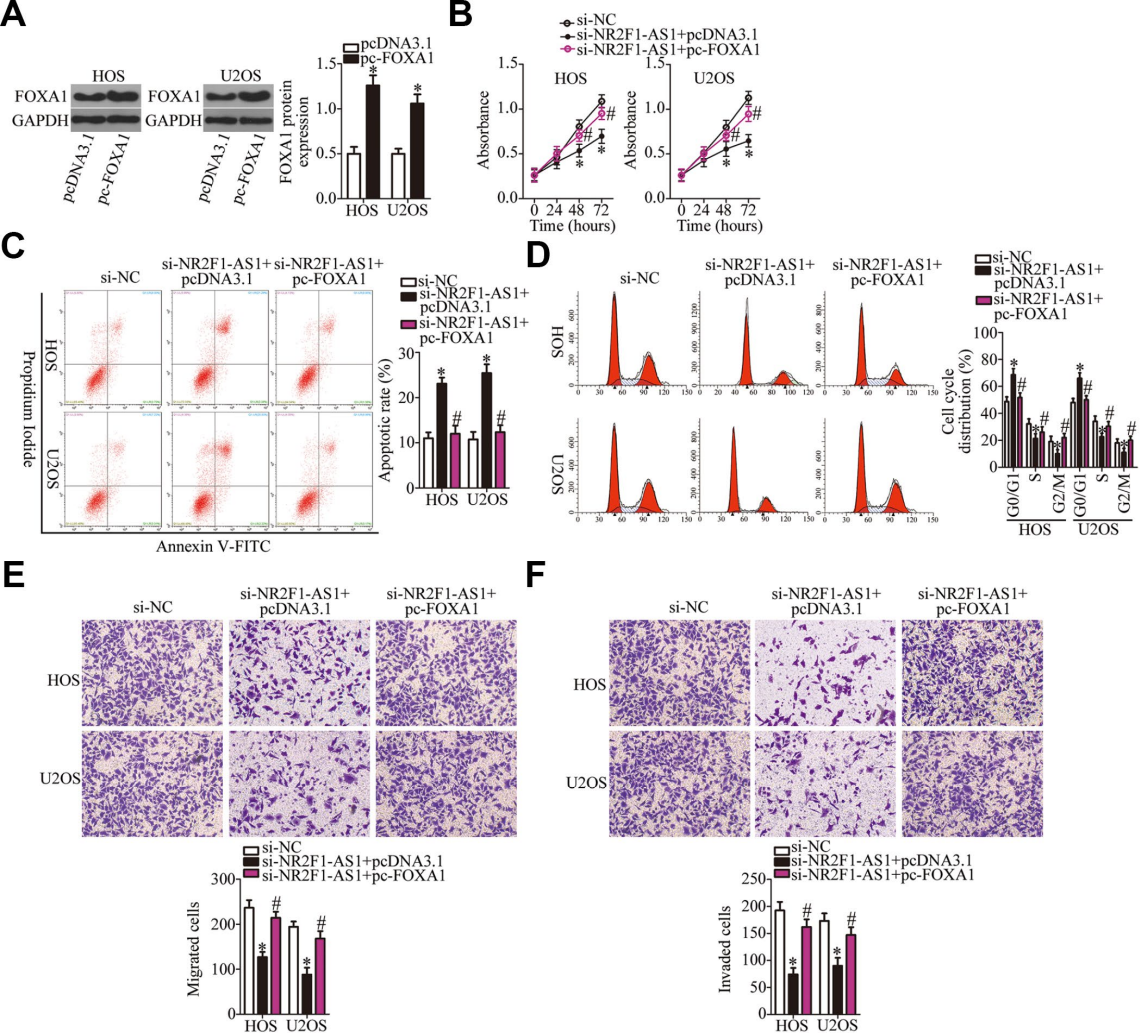
**The actions of the *NR2F1-AS1* knockdown in OS cells are neutralized by recovery of FOXA1.** (**A**) The efficiency of pc-FOXA1 transfection was assessed via western blotting. *P < 0.05 vs. group pcDNA3.1 (empty-vector control). (**B**–**F**) HOS and U2OS cells were cotransfected with si-NR2F1-AS1 and either pc-FOXA1 or the empty pcDNA3.1 vector. The CCK-8 assay, flow cytometry, and Transwell migration and invasion assays were conducted to quantitate cellular proliferation, apoptosis, cell cycle distribution, migration, and invasion. *P < 0.05 vs. group si-NC. ^#^P < 0.05 vs. group si-NR2F1-AS1+pcDNA3.1.

Similarly, rescue experiments were performed on HOS and U2OS cells after cotransfection with si-NR2F1-AS1 and either pc-FOXA1 or the empty pcDNA3.1 vector. Western blotting revealed that transfection with pc-FOXA1 successfully increased the protein amount of FOXA1 (Figure 6A, P < 0.05). Functional experiments on cell proliferation, apoptosis, cell cycle, migration, and invasion were conducted next, and the results demonstrated that the *NR2F1-AS1* knockdown–mediated effects on HOS and U2OS cell proliferation (Figure 6B, P < 0.05), apoptosis (Figure 6C, P < 0.05), cell cycle (Figure 6D, P < 0.05), migration (Figure 6E, P < 0.05), and invasion (Figure 6F, P < 0.05) were substantially attenuated by the recovery of FOXA1. In summary, our data suggested that the miR-483-3p–FOXA1 axis is the mediator of *NR2F1-AS1* functions in OS cells.

### *NR2F1-AS1* knockdown impairs tumor growth *in vivo*

**Figure 7:**
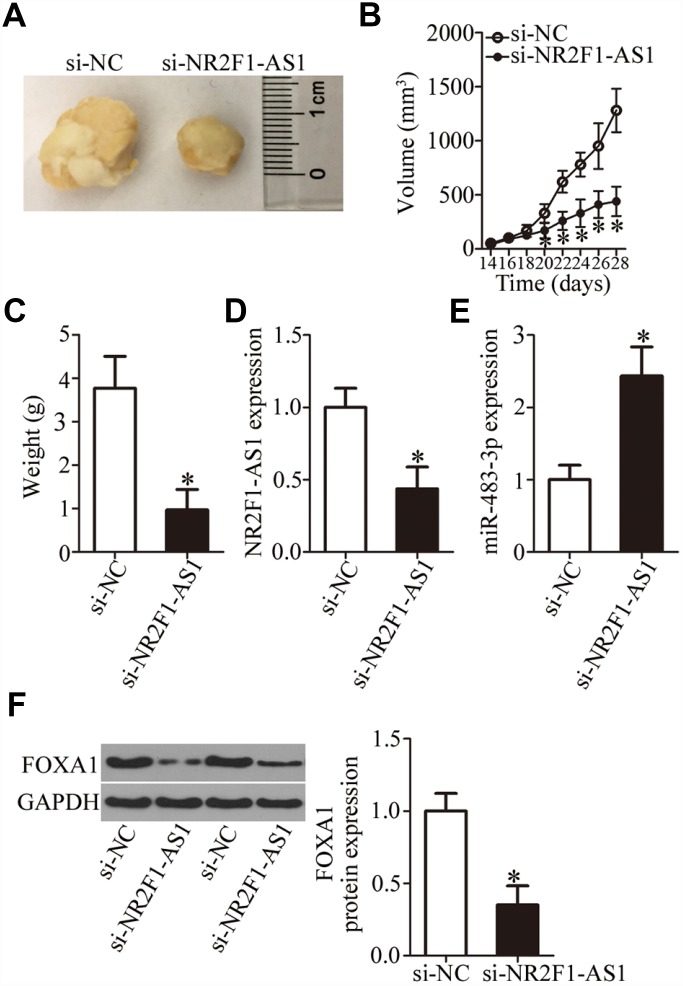
***NR2F1-AS1* knockdown restrains the tumor growth of OS cells *in vivo*.** (**A**) Representative images of tumor xenografts derived from HOS cells transfected with either si-NR2F1-AS1 or si-NC. (**B**) The volume of tumor xenografts derived from si-NR2F1-AS1–transfected or si-NC–transected HOS cells at 4 weeks postinjection. The tumor growth curves were constructed accordingly. *P < 0.05 vs. the si-NC group. (**C**) Weights of tumor xenografts. *P < 0.05 vs. group si-NC. (**D**, **E**) The expression levels of *NR2F1-AS1* and miR-483-3p were determined in the tumor xenografts by RT-qPCR. *P < 0.05 vs. group si-NC. (**F**) Western blotting was conducted to measure FOXA1 protein amounts in the tumor xenografts from the si-NR2F1-AS1 group and si-NC group. *P < 0.05 vs. the si-NC group.

Having clarified the oncogenic roles of *NR2F1-AS1* in OS cells *in vitro*, we next studied the functions of *NR2F1-AS1* *in vivo*. To this end, a tumor xenograft experiment was conducted to test whether the *in vivo* effect was consistent with our *in vitro* observations. HOS cells transfected with si-NR2F1-AS1 were inoculated into mice. At 4 weeks post injection, all the mice were euthanized and the tumor xenografts were excised. A representative image of the resultant tumor xenografts is provided in Figure 7A. The si-NR2F1-AS1 group manifested significantly slower growth *in vivo* as compared to the si-NC group (Figure 7B, P < 0.05). In addition, the weight of tumor xenografts was found to be lower in the si-NR2F1-AS1 group than in the si-NC group (Figure 7C, P < 0.05). The expression of *NR2F1-AS1*, miR-483-3p, and FOXA1 protein in the tumor xenografts was measured next. *NR2F1-AS1* expression was lower (Figure 7D, P < 0.05), while miR-483-3p expression was higher (Figure 7E, P < 0.05) in the tumor xenografts derived from si-NR2F1-AS1–transfected HOS cells, as evidenced by RT-qPCR analysis. In addition, tumor xenografts derived from si-NR2F1-AS1–transfected HOS cells featured lower FOXA1 protein expression compared with the si-NC group (Figure 7F, P < 0.05). These results suggested that *NR2F1-AS1* knockdown retarded the tumor growth of OS cells *in vivo*, and that this phenomenon is mediated by the miR-483-3p–FOXA1 axis.

## DISCUSSION

Numerous lncRNAs are dysregulated in OS, and this aberration serves as the main driver of OS initiation and progression by changing the regulation of a variety of cancer-related biological events [31–33]. In addition, lncRNAs are regarded as novel diagnostic and prognostic biomarkers as well as promising targets for anticancer therapy [34]. Hence, studying the specific involvement of lncRNAs in OS and revealing their mechanisms of action may facilitate the identification of effective therapeutic targets and the development of diagnostic techniques for patients with OS. In this study, we measured *NR2F1-AS1* expression in OS and determined the clinical and prognostic value of *NR2F1-AS1* in OS. In addition, a loss-of-function assay was carried out to assess the impact of *NR2F1-AS1* on the malignant properties of OS cells *in vitro* and *in vivo*. A series of molecular biological experiments was conducted to investigate the mechanism underlying the oncogenic properties of *NR2F1-AS1* in OS.

*NR2F1-AS1* is overexpressed in hepatocellular carcinoma tissues and cell lines [26]. Downregulation of *NR2F1-AS1* inhibits hepatocellular carcinoma cell migration and invasion as well as expression of a drug resistance gene and decreases the half-maximal inhibitory concentration (IC_50_) *in vitro*; *NR2F1-AS1* knockdown also impairs tumor growth *in vivo* via the miR-363–ABCC1 axis [26]. *NR2F1-AS1* is also highly expressed in endometrial cancer [27]. *NR2F1-AS1* knockdown reduces cancer cell viability and metastasis and promotes apoptosis; these actions are mediated by the sponging of miR-363 and increased *SOX4* expression, resulting in the activation of the PI3K–AKT–GSK-3β pathway [27]. Nonetheless, neither the expression profile nor the function of *NR2F1-AS1* in OS have been reported previously. We found that *NR2F1-AS1* is upregulated in OS tissue samples and cell lines. The high *NR2F1-AS1* expression significantly correlated with more advanced clinical stage and distant metastasis among our 53 patients with OS. Patients with OS in the *NR2F1-AS1* high-expression group demonstrated shorter overall survival than did the patients in the *NR2F1-AS1* low-expression group. *NR2F1-AS1* knockdown attenuated OS cell proliferation, migration, and invasion and promoted apoptosis *in vitro* as well as slowed tumor growth *in vivo*.

To gain a fuller understanding of the oncogenic actions of *NR2F1-AS1* in OS progression, a series of experiments was conducted to investigate its regulatory mechanism in detail. First, a nuclear/cytoplasmic fractionation assay showed that *NR2F1-AS1* is mainly located in the cytoplasm of OS cells, suggesting that this lncRNA exerts its effects in OS by interacting with miRNA. Second, bioinformatics analysis identified miR-483-3p as a predicted target of *NR2F1-AS1*. Third, luciferase reporter assays uncovered a direct interaction between miR-483-3p and *NR2F1-AS1* in OS cells. Fourth, a RIP assay confirmed that miR-483-3p is a target of *NR2F1-AS1*. Fifth, miR-483-3p expression was found to be low in OS tissues, with its expression inversely correlated with that of *NR2F1-AS1*. Sixth, depletion of *NR2F1-AS1* notably increased miR-483-3p expression and decreased FOXA1 expression in OS cells. Finally, miR-483-3p knockdown and FOXA1 reintroduction counteracted the effects of the *NR2F1-AS1* knockdown on the malignant behaviors of OS cells. These observations collectively imply that *NR2F1-AS1* serves as a ceRNA of miR-483-3p and thereby positively regulates FOXA1 expression in OS cells.

MiR-483-3p is underexpressed in breast cancer [28, 29], colon cancer [30], and gastric cancer [30]. In our study, we also demonstrated that miR-483-3p is downregulated in OS tissues and cell lines. Functionally, miR-483-3p was validated as a tumor-suppressive miRNA in relation to OS progression and was found to be involved in the regulation of multiple aggressive characteristics. Investigation of the mechanism involved showed that *FOXA1* is a direct target gene of miR-483-3p in OS cells. FOXA1, a member of the forkhead transcription factor family, is closely related to the formation and progression of many human cancers, including OS [35–37]. Our current study indicates that the knockdown of *NR2F1-AS1* reduces the sponging of miR-483-3p, thus reducing FOXA1 expression in OS and suppressing the malignancy of OS cells *in vitro* and *in vivo*. Therefore, our results suggest that targeting the *NR2F1-AS1*–miR-483-3p–FOXA1 pathway may have implications for the treatment of OS.

Overall, this study suggests that *NR2F1-AS1* is upregulated in OS, and its upregulation is related to worse clinical parameters and shorter overall survival among patients with OS. Moreover, *NR2F1-AS1* knockdown decreases the expression of FOXA1 in OS cells by attenuating the sponging of miR-483-3p by *NR2F1-AS1* as its ceRNA. The *NR2F1-AS1*–miR-483-3p–FOXA1 regulatory network is implicated in the aggressiveness of OS cells *in vitro* and *in vivo*.

## MATERIALS AND METHODS

### Ethics statement

Ethical approval was obtained from the Ethics Committee of Liaoning Cancer Hospital and Institute, and written informed consent was provided by all the participants. This study was conducted in compliance with the principles of the Declaration of Helsinki. All the animal experiments were approved by the Institutional Animal Care and Use Committee of China Medical University and were conducted in accordance with the Animal Protection Law of the People’s Republic of China-2009 for experimental animals.

### Clinical samples

OS tissue samples and the adjacent normal tissues were obtained from 53 patients with OS who were admitted to Liaoning Cancer Hospital and Institute between February 2013 to March 2014. These patients had not been treated with preoperative chemotherapy or radiotherapy. All the patients were followed-up for 5 years. The collected tissue samples were quickly placed in storage in liquid nitrogen for further analysis.

### Cell lines and culture conditions

Human OS cell lines (HOS, U2OS, MG-63, and SAOS-2) and human normal osteoblasts (hFOB1.19) were purchased from the Shanghai Institute of Biochemistry and Cell Biology (Shanghai, China). The cells were cultured in Dulbecco’s Modified Eagle’s Medium (DMEM) supplemented with 10% of fetal bovine serum (FBS) and 1% of a penicillin/streptomycin solution (all from Gibco, Thermo Fisher Scientific, Inc., Waltham, MA, USA). The cells were grown at 37°C in a humidified atmosphere containing 5% CO_2_.

### Oligonucleotides and transient transfection

Small interfering RNA (siRNA) against *NR2F1-AS1* (si-NR2F1-AS1) and negative control (NC) siRNA (si-NC) were provided by RiboBio Co., Ltd. (Guangzhou, China). MiR-483-3p agomir (agomir-483-3p), agomir-NC, antagomir-483-3p, and antagomir-NC were chemically synthesized by GenePharma Co., Ltd. (Shanghai, China). A FOXA1 overexpression plasmid pcDNA3.1-FOXA1 (pc-FOXA1) and empty pcDNA3.1 vector were purchased from GeneChem (Shanghai, China). For transfection, cells were seeded in 6-well plates. All the siRNA (100 pmol), agomir (50 nM), antagomir (100 nM) and/or plasmids (4 μg) were transfected into the cells using Lipofectamine® 2000 reagent (Invitrogen; Thermo Fisher Scientific, Inc.).

### RNA isolation and reverse-transcription quantitative polymerase chain reaction (RT-qPCR)

The isolation of total RNA was carried out using TRIzol® reagent (Invitrogen; Thermo Fisher Scientific, Inc.). The quantity and quality of the isolated total RNA were evaluated on a NanoDrop™ 2000 Spectrophotometer (NanoDrop; Thermo Fisher Scientific, Inc.). To analyze *FOXA1* mRNA and *NR2F1-AS1* expression, cDNA was synthesized from total RNA via the PrimeScript RT-Reagent Kit (Takara Bio, Kusatsu, Japan). The synthesized cDNA was then subjected to qPCR with the SYBR Premix Ex Taq™ Kit (Takara Bio), with *GAPDH* as an internal reference. The qPCR was performed with cycling conditions as follows: 5 min at 95°C, followed by 40 cycles of 95°C for 30 sec and 65°C for 45 sec.

For the measurement of miR-483-3p expression, the miRcute miRNA First-Strand cDNA Synthesis Kit (Tiangen Biotech, Beijing, China) was utilized for reverse transcription. After that, miRcute miRNA qPCR Detection Kit SYBR Green (Tiangen Biotech) was employed for PCR amplification. The thermocycling conditios for qPCR were as follows: 95°C for 2 min, 95°C for 10 sec, 55°C for 30 sec and 72°C for 30 sec, for 40 cycles. U6 small nuclear RNA served as the control for miR-483-3p expression analysis. All the reactions were carried out on an ABI 7500 System (Applied Biosystems; Thermo Fisher Scientific Inc.). Relative expression of *FOXA1*, *NR2F1-AS1,* and miR-483-3p were calculated by the 2^−ΔΔCq^ method.

The primers were designed as follows: NR2F1-AS1, 5′-CAGCGGTGCAAACCATGTGC-3′ (forward) and 5′-GTAAACCAAGTCGGTTGAACG-3′ (reverse); FOXA1, 5′-CGCTTCGCACAGGGCTGGAT-3′ (forward) and 5′-TGCTGACCGGGACGGAGGAG-3′ (reverse); GAPDH, 5′-CGGAGTCAACGGATTTGGTCGTAT-3′ (forward) and 5′-AGCCTTCTCCATGGTGGTGAAG AC-3′ (reverse); miR-483-3p, 5′-TCACTCCTCTCCT CCCG-3′ (forward) and 5′-GTGCAGGGTCCGAGGT-3′ (reverse); and U6, 5′-TGCGGGTGCTCGCTTCGGC AGC-3′ (forward) and 5′-CCAGTGCAGGGTCCG AGGT-3′ (reverse).

### Cell Counting Kit-8 (CCK-8) assay

Transfected cells were collected at 24 h post transfection, and single-cell suspensions were prepared. In total, 100 μL of a cell suspension containing 2 × 10^3^ cells was added into each well of the 96-well plates. The CCK-8 assay was performed to determine cellular proliferation. Briefly, into every well we added 10 μL of the CCK-8 solution (Dojindo Laboratories, Kumamoto, Japan) prior to incubation at 37°C for 2 h. Cellular proliferation was assessed by quantitation of absorbance at 450 nm wavelength on an Epoch Microplate Spectrophotometer (BioTek, Winooski, VT, USA). The CCK-8 assay was carried out at four time points—0, 24, 48, and 72 h after cell seeding—and growth curves were constructed based on means of the time points (X-axis) and absorbance values (Y-axis).

### Flow-cytometric analysis of apoptosis and cell cycle distribution

Transfected cells were collected and subjected to the following experimental procedures: they were washed twice with ice-cold phosphate-buffered saline (PBS), centrifuged, resuspended in 100 μl of binding buffer, and double-stained with 5 μl of annexin V–fluorescein isothiocyanate (FITC) conjugate and 5 μl of propidium iodide solution from an Annexin V–FITC Apoptosis Detection Kit (Biolegend, San Diego, CA, USA). After 15 min of incubation at room temperature in darkness, the proportion of apoptotic cells was examined on a flow cytometer (FACScan™; BD Biosciences, Franklin Lakes, NJ, USA).

The cells that underwent 48 h of transfection were immediately collected with EDTA-free trypsin (Gibco, Thermo Fisher Scientific, Inc.) and then washed with cold PBS. After fixation in 70% ethanol at 4°C for 1 h, the transfected cells were treated with 50 μl of RNase (100 μg/ml) at room temperature for 20 min, followed by staining with 25 μl of the propidium iodide solution diluted in 425 μl of cell staining buffer (both from Biolegend). Finally, the cell cycle distribution was determined by flow cytometry after 20 min of incubation at room temperature.

### Transwell migration and invasion assays

After 24 h of incubation, transfected cells were harvested and washed with PBS (Gibco, Thermo Fisher Scientific, Inc.). FBS-free DMEM was used to prepare single-cell suspensions. For the migration assay, 100 μL of a cell suspension containing 5 × 10^4^ cells was placed into the upper compartments of the transwell inserts (BD Biosciences). For the invasion assay, the same number of cells was inoculated into Matrigel (BD Biosciences)-coated upper compartments. In both assays, the lower compartments were filled with DMEM containing 20% of FBS. The cells were then maintained at 37°C in a humidified atmosphere containing 5% of CO_2_ for 24 h and then fixed with 4% paraformaldehyde and stained with 0.5% crystal violet. After extensive washing, the stained cells were imaged under an Olympus microscope (x200 magnification; Olympus Corporation, Tokyo, Japan). The migratory and invading cells in at least five randomly selected visual fields were counted, and the data were analyzed.

### A tumor xenograft experiment

Male 4-week-old nude BALB/c mice were bought from the Shanghai Laboratory Animal Center (Chinese Academy of Sciences, Shanghai, China) and were bred under specific pathogen-free conditions. Equal numbers of HOS cells (1 × 10^7^) transfected with either si-NR2F1-AS1 or si-NC were collected, resuspended in 100 μL of FBS-free DMEM, and subcutaneously injected into a flank of each mouse (n = 4 for each group). The volume of the formed tumor xenografts was recorded every 2 days via the following formula: tumor volume = ½ × tumor length × tumor width^2^. After 4 weeks, all the mice were euthanized through cervical dislocation. The tumor xenografts were excised, weighed, and subjected to total-RNA and total-protein isolation.

### Nuclear/cytoplasmic fractionation

A PARIS Kit (Invitrogen; Thermo Fisher Scientific, Inc.) was employed to isolate cytoplasmic and nuclear fractions.

### RNA immunoprecipitation (RIP) assay

This assay was carried out using the Magna RIP RNA-Binding Protein Immunoprecipitation Kit (Millipore Inc., Billerica, MA, USA). Transfected cells were lysed in RIPA buffer that was supplemented with an RNase inhibitor and protease inhibitor cocktail. After that, the cell extract was probed with magnetic beads conjugated with a human anti-AGO2 antibody (Millipore) or IgG control (Millipore Inc.). After protein digestion, immunoprecipitated RNA was obtained and subjected to RT-qPCR analysis.

### Bioinformatics prediction and luciferase reporter assay

The miRNA(s) that may be sponged by *NR2F1-AS1* was predicted using starBase 3.0 (http://starbase.sysu.edu.cn/). Two bioinformatics tools, namely, miRDB (http://mirdb.org/), and TargetScan (http://www.targetscan.org/), were utilized for searching for the putative target(s) of miR-483-3p.

*NR2F1-AS1* cDNA containing the predicted wild type (wt) miR-483-3p–binding sequences was amplified by Shanghai GenePharma Co., Ltd., and inserted into the pmirGLO luciferase reporter vector (Promega, Madison, WI, USA), resulting in a plasmid designated as NR2F1-AS1-wt. Plasmids NR2F1-AS1-mutant (mut), FOXA1-wt, and FOXA1-mut were constructed in the same way. Either wt or mut reporter plasmids plus either agomir-483-3p or agomir-NC were cotransfected into cells using the Lipofectamine® 2000 reagent. The luciferase activity in the transfected cells was quantified with a Dual-Luciferase Reporter System (Promega). Firefly luciferase activity was normalized to that of Renilla luciferase.

### Western blot analysis

Total cellular protein was isolated with RIPA buffer containing protease and phosphatase inhibitors. After centrifugation for 5 min at 4°C, the supernatant was collected and subjected to the quantification of total protein by the Bicinchoninic Acid Assay (Beyotime Institute of Biotechnology, Inc., Shanghai, China). Equal amounts of protein were separated by SDS-PAGE in 10% polyacrylamide gels and transferred to polyvinylidene fluoride membranes (Millipore Inc.). Prior to overnight incubation with primary antibodies, the membranes were blocked with 5% nonfat milk at room temperature for 2 h. The membranes were rinsed with Tris-buffered saline containing 0.1% of Tween 20 (TBST) thrice and next incubated at room temperature for 1 h with a horseradish peroxidase–conjugated secondary antibody (ab205718; 1:5000 dilution in TBST; Abcam, Cambridge, UK, USA). Finally, the protein signals were detected by means of the SuperSignal West Pico Chemiluminescent Substrate Kit (Pierce; Thermo Fisher Scientific, Inc.). Antibodies against FOXA1 (ab170933; Abcam) and GAPDH (ab128915; Abcam) (primary antibodies) were acquired from Abcam and were applied at 1:1000 dilution in TBST. Quantity One software version 4.62 (Bio Rad Laboratories, Inc., Hercules, CA, USA) was applied to analyze the densitometry.

### Statistical analysis

All experiments were repeated three times, and all data were formatted as the mean ± standard deviation. All statistical analyses were conducted using SPSS software (version 19.0; IBM Corp., Armonk, NY, USA). The associations between *NR2F1-AS1* expression and clinical parameters of the patients with OS were determined via the χ^2^ test. Correlations of expression levels between two genes (*NR2F1-AS1* vs *MIR-483-3p*, *MIR-483-3p* vs *FOXA1*) among the OS tissue samples were studied by Spearman’s correlation analysis. The Kaplan–Meier method was applied to assess the overall survival of patients with OS. The survival distribution between groups was assessed by the log rank test. The differences between two groups and among multiple groups were evaluated with Student’s *t* test and one-way analysis of variance (ANOVA), respectively, followed by the Student–Newman–Keuls *post hoc* test. P < 0.05 was assumed to indicate a statistically significant difference.
